# Identification of Cherry Tomato Volatiles Using Different Electron Ionization Energy Levels

**DOI:** 10.3390/molecules29235567

**Published:** 2024-11-25

**Authors:** Dalma Radványi, László Csambalik, Dorina Szakál, Attila Gere

**Affiliations:** 1 Department of Hospitality, Faculty of Commerce, Hospitality and Tourism, Budapest Business University, 9-11 Alkotmány út, H-1054 Budapest, Hungary; gerene.radvanyi.dalma@uni-bge.hu (D.R.);; 2 Department of Agroecology and Organic Farming, Institute of Sustainable Development and Economics, Hungarian University of Agricultural and Life Sciences, 29-43 Villányi út, H-1118 Budapest, Hungary; 3 Department of Postharvest Science, Trade, Supply Chain and Sensory Evaluation, Institute of Food Science and Technology, Hungarian University of Agricultural and Life Sciences, 29-43 Villányi út, H-1118 Budapest, Hungary

**Keywords:** tomato volatiles, HS-SPME-GC-MS, GC-MS, EI, cherry tomato, unknown analysis

## Abstract

A comprehensive analysis of the volatile components of 11 different cherry tomato pastes (Tesco Extra, Orange, Zebra, Yellow, Round Netherland, Mini San Marzano, Spar truss, Tesco Sunstream, Paprikakertész, Mc Dreamy, and Tesco Eat Fresh) commercially available in Hungary was performed. In order to ensure the reliability and accuracy of the measurement, the optimal measurement conditions were first determined. SPME (solid-phase microextraction) fiber coating, cherry tomato paste treatment, and SPME sampling time and temperature were optimized. CAR/PDMS (carboxen/polydimethylsiloxane) fiber coating with a film thickness of 85 µm is suggested at a 60 °C sampling temperature and 30 min extraction time. A total of 64 common compounds was found in the prepared, mashed cherry tomato samples, in which 59 compounds were successfully identified. Besides the already published compounds, new, cherry tomato-related compounds were found, such as 3 methyl 2 butenal, heptenal, *Z*-4-heptenal, *E*-2-heptenal, *E*-carveol, verbenol, limonene oxide, 2-decen-1-ol, *Z*-4-decen-1-al, caryophyllene oxide, and *E*,*E*-2,4-dodecadienal. Supervised and unsupervised classification methods have been used to classify the tomato varieties based on their volatiles, which identified 16 key components that enable the discrimination of the samples with a high accuracy.

## 1. Introduction

Tomatoes (*Solanum lycopersicum*) are one of the most widely cultivated and consumed fruits in the world. While in 2021 tomato production was 189 million tonnes, in 2022 it was estimated at 186 million tonnes, of which 20 million tonnes were produced in the European Union (EU). Although tomatoes are not among the top 10 most-produced commodities in Hungary, tomato production is not negligible, with 137,860 tonnes of tomatoes being produced in 2022 [1].

Tomatoes are rich in lycopene, vitamin C, minerals, and many other essential nutrients, making them of considerable economic importance [2,3,4]. Tomato is a highly diverse species with a wide range of cultivars exhibiting significant variability in size, shape, color, flavor, and even aroma [5,6,7]. This diversity results from extensive breeding and domestication, aimed at enhancing specific traits to meet consumer preferences and agricultural demands. Tomatoes can be broadly differentiated based on their size. Cherry tomatoes are the smallest variety, typically measuring 1–2 cm in diameter and these tomatoes are known for their sweet flavor due to their high sugar content and juicy texture [8]. Plum (or Roma) tomatoes are medium-sized, typically 5–7 cm, and have an elongated shape. Beefsteak tomatoes are the largest variety, often weighing over 500 g each. Tomato varieties also exhibit a remarkable range of colors, each contributing unique visual appeal and potential nutritional benefits. Red tomatoes are the most common and are rich in lycopene, a potent antioxidant linked to various health benefits. The red color is primarily due to the high concentration of lycopene. Yellow and orange tomatoes are typically milder and less acidic than red tomatoes. They contain different carotenoids, such as beta-carotene, which the body can convert to vitamin A. Green tomatoes can be either unripe fruits of any variety or specific cultivars that remain green when ripe. Purple and black tomatoes have a rich, complex flavor profile with earthy and smoky undertones. Their dark color is due to the presence of anthocyanins, which have antioxidant properties [5,9,10,11,12,13].

In addition to size and color, tomatoes can be distinguished by various other characteristics, including flavor (taste and aroma). Flavor is a critical quality attribute for tomatoes, significantly influencing consumer preference and marketability. Tomato flavor is a complex property, which derives from the interaction of sugars, acids, and several volatile organic compounds (VOCs). Sugars (mainly glucose and fructose) contribute sweetness, while organic acids (such as citric or malic acids) add the acidity. Volatile compounds play a key role in the aroma profile. Cherry tomatoes’ volatile profile includes fruity esters, aldehydes, and alcohols. Key volatiles in cherry tomatoes often include hexanal, (*E*)-2-hexenal, and 6-methyl-5-hepten-2-one, which contribute to their fresh, green, and slightly floral aroma. The presence and concentration of these volatiles can vary widely among different tomato varieties, influenced by genetic factors, growing conditions, and post-harvest handling. Beefsteak tomatoes have a volatile profile, which tends to include higher levels of terpenes and norisoprenoids, such as β-ionone and geranylacetone, which contribute to their complex aroma. Plum tomatoes’ volatile profile is characterized by a higher concentration of ketones and aldehydes that withstand heat and maintain flavor during cooking [14]. Compounds, such as 1-penten-3-one and 3-methylbutanal, are prominent in plum tomatoes. Heirloom tomatoes often have a richer and more complex aroma, including a broader range of esters, alcohols, and sulfur compounds. Volatiles, such as 2-isobutylthiazole and 2-phenylethanol, are commonly found in heirloom tomatoes, providing distinctive earthy, floral, and fruity notes [10,15]. Mayer and his coworkers (2008) built an aroma model of a tomato, where 1-penten-3-one,(*E*,*E*); (*E*,*Z*)-2,4-decadienal; 4-hydroxy-2,5-dimethyl-3(2*H*)-furanone (furaneol); 2-phenylethanol; and 2-isobutylthiazole were the key compounds [16]. Understanding and optimizing the profile of these volatile compounds are therefore essential for breeders and the food industry to enhance the quality of tomatoes [17,18].

Monitoring volatile changes can help determine the optimal harvest time and ensure that tomatoes reach consumers at their peak flavor. Volatiles can also be used as a freshness indicator helping to optimize storage conditions and improve shelf life. Moreover, food adulteration can also be detected by the measurement of volatile compounds [19]. Scientific studies have demonstrated that the volatile profiles of different tomatoes can be used to distinguish different cultivars, ripeness stages, growing conditions, regions, and even food adulteration. The unique composition and concentration of volatile organic compounds (VOCs) in each tomato variety contribute to its distinctive aroma and flavor [15,20,21].

Near-infrared (NIR) spectroscopy and electronic tongue (e-tongue) are already known as advanced, high-sensitivity techniques for quality control [22], but volatile compounds can also be used for detecting adulteration in the case of tomato paste [23]. Several analytical techniques are available to measure volatiles, in our case, tomato paste volatiles. These techniques range from conventional gas chromatography to advanced mass spectrometry, each offering specific advantages in terms of sensitivity, resolution, and the ability to identify and quantify a wide range of volatiles. Gas chromatography (GC) is one of the most widely used techniques for volatile analysis. It involves the separation of volatile compounds based on their volatilities and interactions with the stationary phase of the chromatography column. Combining gas chromatography with mass spectrometry (GC-MS) allows for the identification and quantification of a broad range of volatiles, even unknown volatiles, thanks to spectrum libraries because they provide detailed information about the molecular structure of the compounds. The SPME (solid-phase microextraction) sample preparation technique involves the adsorption of volatile compounds onto a coated fiber, which is then desorbed in the injection port of a GC. It is a solvent-free method that is particularly useful for analyzing complex matrices, like tomato tissues. This sampling technique can be easily coupled with GC-MS without direct contact with the sample. An electronic nose (E-nose) is a sensor-based technology that mimics the human olfactory system. It uses an array of sensors to detect and differentiate volatile compounds, providing a rapid and non-destructive method for assessing tomato aroma. E-nose technology is also useful for the quick screening and differentiation of samples in quality control settings [4,10,15,21,24,25,26]. While the HS-SPME-GC-MS technique provides detailed data, it is linked to the laboratory due to its size and technical specifications. Novel applications of e-nose focus on the development of portable e-nose systems coupled with advanced chemometric tools that enable researchers to complete field measurements easily [27].

The main objective of this study was to use the HS-SPME-GC-MS method for capturing and analyzing tomato volatiles and successfully identifying unknown volatiles. As a result, we aim to develop a suitable and green solution for unknown volatile compound identification without standard solutions. Our secondary aim was to distinguish different commercially available mashed cherry tomatoes based only on their VOCs to create an approach for the detection of food adulteration or quality changes.

## 2. Results

### 2.1. Sampling Optimization

The appropriate optimization of sampling is essential during measurements [28]. Therefore, in the first step, SPME fiber coating, sample treatment, and SPME sampling time and temperature were optimized.

**Figure 1:**
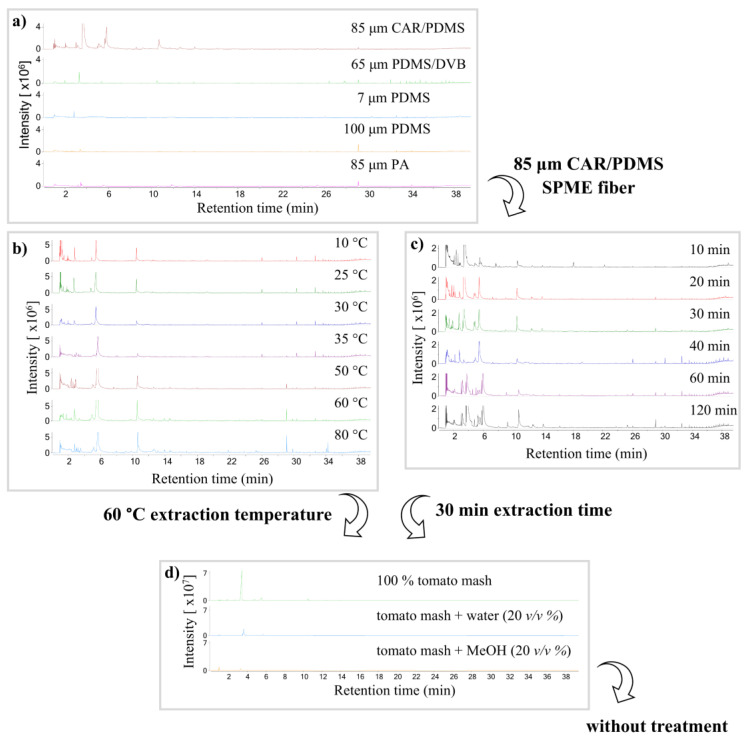
(**a**) Comparison of SPME fiber coatings in the case of captured tomato volatiles. (**b**) Examination of the effect of seven different extraction temperatures (10, 25, 30, 35, 50, 60, and 80 min) on captured VOCs from tomatoes. (**c**) Examination of the effect of six different extraction times (10, 20, 30, 40, 60, and 120 min) on captured VOCs from tomatoes. (**d**) Evaluation of the effect of solvent addition (methanol and water).

As a first step, five different SPME fibers were used for sampling optimization: CAR/PDMS (carboxen/polydimethylsiloxane, 85 µm), PA (polyacrylate, 85 µm), PDMS (polydimethylsiloxane, 7 µm and 100 µm), and PDMS/DVB (polydimethylsiloxane/divinylbenzene, 65 µm) (Supelco™, Bellefonte, PA, USA). Sampling parameters were kept constant during this optimization step; the sampling time was 40 min and the temperature was 60 °C. The CAR/PDMS SPME fiber extracted the most compounds with the highest intensity value (Figure 1a), and most of the volatiles were observed at the beginning of the chromatogram. The PDMS/DVB fiber coating captured volatiles at a higher intensity value at the end of the chromatogram. However, the summary of peak abundance was much higher in the case of CAR/PDMS; therefore, this type of fiber coating was selected. PDMS and PA fibers have a much lower efficiency when capturing tomato volatiles.

The balance between the SPME fiber coating and the sample is a critical factor because the maximum number of compounds can be captured after reaching equilibrium. The extraction time is influenced by several factors, such as extraction type, sample temperature, fiber coating thickness and type, and sample concentration. Since the extraction temperature has a great effect on the efficiency of the SPME volatile extraction method [29], the optimization of the extraction temperature was carried out with the help of a water batch. A previously chosen CAR/PDMS fiber was used at 10, 25, 30, 35, 50, 60, and 80 °C in order to examine the effect of the extraction temperature. The extraction time was 40 min for all measurements. A temperature of 60 °C proved to be the best sampling temperature. At this temperature, chromatographic peaks of appreciable intensity were observed (Figure 1b). Moreover, no special laboratory conditions were required to maintain the temperature.

After temperature optimization, the effect of the extraction time was also investigated. Extraction time plays a key role in allowing the fiber to capture the right amount of volatile organic compounds. Extraction times of 10, 20, 30, 40, 60, and 120 min were examined, and the extraction temperature was set to 60 °C as an optimized extraction temperature. Figure 1c shows that most VOCs can be qualitatively identified at 30 min of sampling. Moreover, equilibrium is achieved for most components in 30 min; therefore, the 30 min extraction time was chosen as an optimal extraction time at 60 °C.

According to the literature, the number of vaporized compounds can be increased by solvent addition. Beltran and his co-workers suggest that small amounts of methanol promote the removal of volatiles from the matrix by facilitating their transfer to the gas phase [30]. Therefore, three different sample treatments were compared: (i) homogenized tomato mash, (ii) homogenized tomato mash diluted with distilled water (20 *v*/*v*%), and (iii) tomato juice diluted with methanol (20 *v*/*v*%). Measurements were carried out in triplicates under a controlled SPME measuring process (the extraction time was 30 min; the extraction temperature was 60 °C). The results indicate that no special sample treatment is needed to collect more volatile compounds from the mashed tomato samples (Figure 1d).

Based on the measured data, a CAR/PDMS fiber with a film thickness of 85 µm, a sampling time of 30 min, and a sampling temperature of 60 °C are the optimal sampling conditions for the measurement of volatile organic compounds in cocktail tomatoes.

### 2.2. Identification of VOCs in Tomato Mash

During the analysis of the total ion chromatogram (TIC), more than 150 volatile compounds were found. For high-intensity compounds, the mass spectrum of the compound was compared with the known mass spectra in the NIST mass spectral library. If the match was above 80%, the identification result was accepted. For the identification of low-intensity compounds, a background correction was applied (i.e., the background mass numbers were subtracted from the mass spectrum of the component), thus improving the accuracy of the identification. A total of 64 compounds was found in the mashed cherry tomato samples. These compounds occurred in all samples, therefore they are the common compounds, which describe the most of the tomato aromas. Among the 64 components, 59 compounds were successfully identified using the NIST mass spectra (MS) library, with at least 80% of the match factor (Table 1). The odor characteristic column shows the smell of some aroma compounds, which may characterize the aroma of tomato [31,32].

Several compounds, such as 1-hexanal, 2-hexenal, *E*-2-hexenal, *E*-3-hexen-1-ol, 1-hexanol, *E*,*E*-2,4-hexadienal, 6-methyl-5-hepten-2-one, *E*,*E*-2,4-nonadienal, 2-isobutylthiazole, phenylacetaldehyde, *E*-2-octenal, *Z*- and *E*-citral, 1-hydroxy-2-methoxy-benzene, benzeneethanol, β-pinene, *E*-2-decenal, *E*,*Z*-2,4-decadienal, R-limonene, geranyl acetone, and β-cyclocitrylideneacetone were found, which have already been described in studies regarding the aroma compounds of tomato volatiles [30,31,33,34].

Additionally, some compounds were found that have not been linked to tomato volatiles to date. Our study adds some new compounds to the list of volatile compounds (such as 3-methyl-2-butenal, heptenal, *Z*-4-heptenal,*E*-2-heptenal, *E*-carveol, verbenol, limonene oxide, 2-decen-1-ol, *Z*-4-decen-1-al, caryophyllene oxide, and *E*,*E*-2,4-dodecadienal) that may better describe the mashed cherry tomato’s volatile compounds.

### 2.3. Validation of Identification

In the case of mass spectrum identification, 80% of the match factor value can mean a good but not always satisfactory result. The appropriate standard is not always available for the analysis of unknown volatile compounds, especially when several unknown compounds are to be identified. Therefore, a new approach was used to validate the identification result.

**Figure 2:**
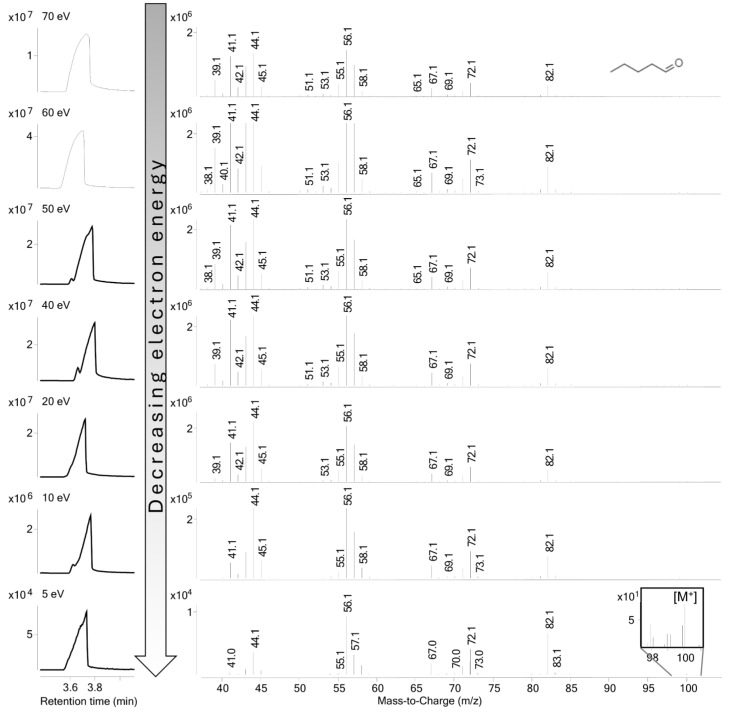
Mass spectra of hexanal at 70 eV, 60 eV, 50 eV, 40 eV, 20 eV, 10 eV, and 5 eV.

An ionization energy value of 70 eV was used to generate characteristic ions of a molecule. At this energy level, minor changes in the electron energy do not significantly affect the pattern of the spectrum, therefore it can be used for building spectrum libraries for organic compounds. By decreasing the electron energy, there is a general loss of intensity due to the decrease in ionization efficiency. However, the lowering of the ionization voltage may favor some fragmentation processes. Moreover, a modification of electron energy may allow us to find the molecular ion [M^+^] if the intensity loss is not significant (Figure 2).

**Figure 3:**
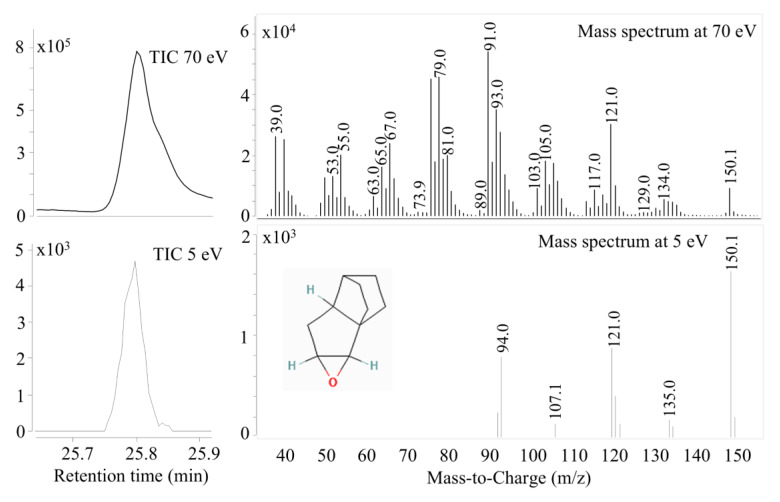
Mass spectra of 2H-1b,4-Ethanopentaleno[1,2-b]oxirene,hexahydro-(1aa,1bb,4b,4aa,5aa) at 70 eV and 5 eV. Match factor: 92.8%, structure: C_10_H_14_O, molecular weight: 150.1, and CAS number: 117221-80-4. TIC: total ion chromatogram.

Changes in mass spectra were not detectable by decreasing the ionization energy until 40 eV. At 20 eV, some small fragment intensity values decreased, and finally, we observed a clearer mass spectrum at 10 and 5 eV due to the loss of low-intensity fragments. The intensity values of fragments also decreased with the decreasing energy level, but the molecular ion could still be found in some cases at a 5 eV energy level. However, reliable molecular ions can be found at a 10 eV electron energy level. There were some compounds in which the molecular ion could be seen more clearly on the mass spectrum (Figure 3).

During the analysis, the level of ionization energy in EI was reduced in several steps. By reducing the electron energy level during ionization, the number of molecular fragments decreases, and then, at sufficiently low energy levels, the molecular ion becomes more visible. The first ion appears when the molecule has emitted only one electron due to the ionization energy. This process does not involve the fragmentation of the molecule. The mass of the molecular ion formed is thus equal to the molecular mass, which differs only by the mass of the electron removed. However, this difference is not significant, and the resolution of the analytical instrument does not necessarily allow this difference to be measured. The mother ion is the molecular ion formed from the original volatile molecule, which is no longer fragmented by the low electron energy level but is charged and can be separated in the analyzer.

Figure 3 shows that a maximum intensity value can be measured by the mass number of 150.1 in the case of the 5 eV ionization energy. The relative intensity also increases significantly compared to the other fragments. The ion that appears is indeed the mother ion of the previously identified compound, so it can be confirmed that the component found is 2H-1b,4-Ethanopentaleno[1,2-b]oxirene,hexahydro-(1aa,1bb,4b,4aa,5aa). Table 2 summarizes the compounds that were successfully validated by this identification method with the use of a 10 eV electron energy level. It was not possible to find the mother ion for all compounds and thus identify the compounds in question, since for some compounds, this low ionization energy proved to be too low to create a molecular ion. However, the mother ion was found for 21 compounds at 10 eV, thus validating the identification result.

Following successful method optimization, we measured the samples and identified common components. This raises the question of whether it is possible to distinguish the 11 mashed tomato samples based on the measured volatile compounds, and which components may be responsible for the separation. We sought to answer this question by the statistical analysis of common volatile compounds.

### 2.4. Chemometric Analysis

The abovementioned results show the volatile composition of each mashed tomato, but further analysis of volatile components will allow us to distinguish between individual mashed tomato samples. Agglomerative hierarchical cluster analysis was used for the separation, and more specifically for the grouping of the samples, as this is a well-established, unsupervised procedure for grouping similar samples, while for supervised classification, linear discriminant analysis with principal component analysis (PCA-LDA) was used.

Cluster analysis (CA) was used as an unsupervised technique to test if there is any pattern in the dataset that groups the mashed tomato samples based only on their volatile patterns. The successful identification of patterns (e.g., clusters) in the dataset suggests that a supervised classification will also provide satisfactory results.

**Figure 4:**
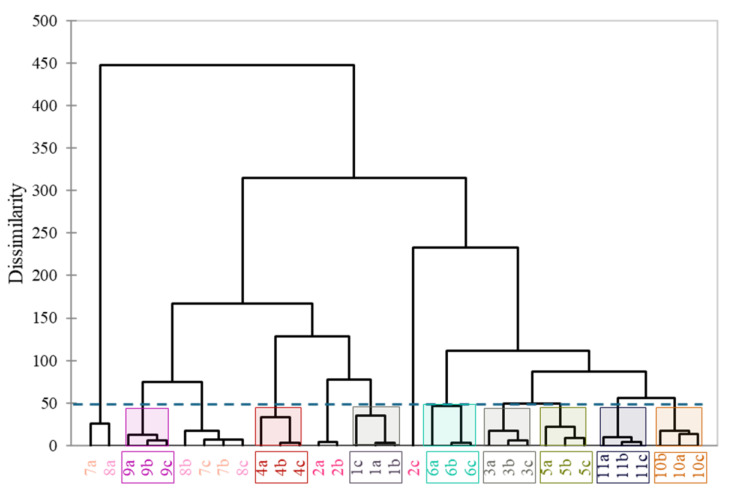
Agglomerative hierarchical clustering using the Euclidean distance and Ward’s method. Dashed line represents an arbitrary cut of the dendrogram. Colors and numbers represent the mashed tomato samples (1: Tesco Extra, 2: Orange, 3: Zebra, 4: Yellow, 5: Round Netherland, 6: Mini San Marzano, 7: Spar truss, 8: Tesco Sunstream, 9: Paprikakertész, 10: Mc Dreamy, and a11: Tesco Eat Fresh), while letters represent the repetitions (a, b, and c).

CA was run using the Euclidean distance and Ward’s method after a careful evaluation of multiple distance measurements and agglomeration schedules as suggested by [35]. This clustering method starts at the lowest level and merges two clusters at each level to form a new cluster. Each level of the hierarchy contains a clustering of the data. The overall hierarchy represents a system of these groups. The results are plotted on a dendrogram (Figure 4).

The dendrogram shows that the majority of the samples has similarities at a low level. Samples 7 and 8 were considered as similar and their first repetitions (7a and 8a) were considered as highly dissimilar to all the other samples. Sample 2c also showed a high dissimilarity compared to 2a and 2b. Other than these, all the other samples are grouped close to each other (sample: 1, 3, 4, 5, 6, 9, 10, and 11), therefore suggesting a strong similarity between the repetitions and a greater difference between the samples. These results also suggest that a classification of the samples is possible, and their volatile compositions bear valuable information.

In order to find out which compounds are involved in grouping the mashed tomato samples, a supervised method was used. Linear discriminant analysis was run on scores obtained using principal component analysis (PCA-LDA) [36]. PCA-LDA enabled the classification of the mashed tomato samples based on their volatiles. PCA was run on the original input matrix and tested using Kaiser–Meyer–Olkin (KMO) and Bartlett’s tests. The Kaiser–Meyer–Olkin measure of sampling adequacy is a statistical measure to determine how suited data are for principal component analysis. This test resulted in a KMO = 0.663, exceeding the limit of 0.6, therefore suggesting that our variables are suitable for PCA. Bartlett’s test of sphericity evaluates whether the correlation coefficients are all 0, proving that the variables correlate with each other. As the test provided significant results (*p* < 0.05), the variables show satisfactory correlations.

**Figure 5:**
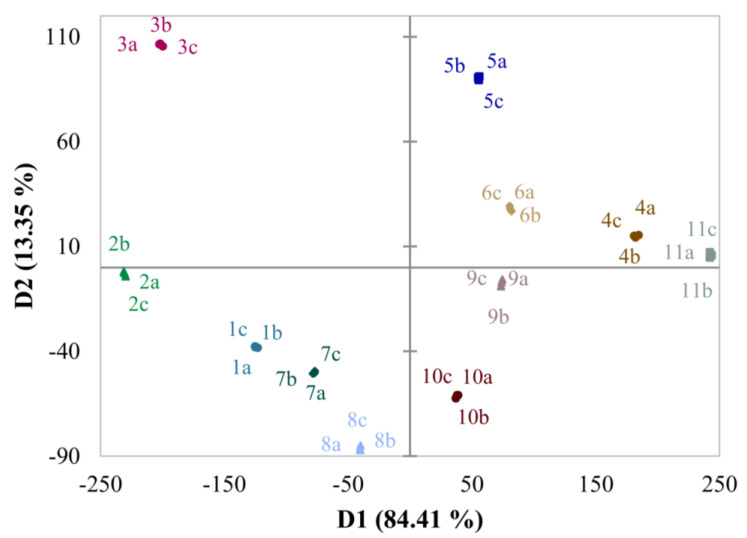
The discriminant analysis runs on principal component analysis loadings. The first discriminant function accounts for 97.76% of variance. Colors and numbers represent the mashed tomato samples (1: Tesco Extra, 2: Orange, 3: Zebra, 4: Yellow, 5: Round Netherland, 6: Mini San Marzano, 7: Spar truss, 8: Tesco Sunstream, 9: Paprikakertész, 10: Mc Dreamy, and 11: Tesco Eat Fresh), while letters represent the repetitions (a, b, and c). D: discriminant function.

Based on the results of AHC, the PCA-LDA provided an acceptable model, as the explained variance of the first two discriminant functions accounts for more than 97% of the variance (Figure 5). The accuracy of the training sample produced 100% accuracy. Leave-one-out cross-validation was performed, where the accuracy was 94.44% with two misses, namely 1b was classified as 7, while 4a was classified as 11.

Altogether, 32 principal components were created that served as the input matrix for the LDA. LDA used forward entering, with a threshold of 0.05. As can be seen in the figure, the samples are sorted into different groups and the separation is successful. As a result, the following 16 compounds were kept in the final model: 2-hexenal; *E*-3-hexen-1-ol; 6-methyl-5-hepten-2-one; 2-isobutylthiazole; 1-oxacyclopropyl-3,4-epoxycyclohexane; 1-hydroxy-2-methoxy-benzene; benzeneethanol; benzyl nitrile; unknown 1 (unidentified C_10_H_16_O compound); verbenol; 2-phenylnitroethane; 2*H*-1b,4-ethanopentaleno[1,2-b]oxirene,hexahydro- (1aa,1bb,4b,4aa,5aa)-; 1,2-15,16-diepoxyhexadecane; caryophyllene oxide; and three unknown compounds (t_R_ = 19.5 min (unidentified C_10_H_16_O compound), and t_R_ = 26.0 min and t_R_ = 30.2 min). According to the results, evaluating 16 compounds instead of 64 is enough for successful separation.

## 3. Discussion

PDMS and PA fiber coatings have a lower efficiency in capturing tomato volatiles. Compounds captured by the PDMS/DVB fiber coating showed higher intensity values at the end of the chromatogram, however the summary of peak abundance was much higher in the case of CAR/PDMS. Therefore, the CAR/PDMS fiber coating with a film thickness of 85 µm is suggested for measurements. While the balance between the SPME fiber coating and the sample is a critical factor for efficient extraction, we found that a 60 °C sampling temperature for a 30 min extraction time provided the highest intensities. It has also been proved that no special sample treatment, like water or methanol addition, was needed to collect the proper amount of volatile compounds from the cherry tomato samples.

A total of 64 common compounds was found in the prepared, mashed cherry tomato samples, in which 59 compounds were successfully identified with the NIST MS mass spectrum library. Several compounds, such as 1-hexanal, 2-hexenal, *E*-2-hexenal, *E*-3-hexen-1-ol, 1-hexanol, *E*,*E*-2,4-hexadienal, 6-methyl-5-hepten-2-one, *E*,*E*-2,4-nonadienal, 2-isobutylthiazole, phenylacetaldehyde, *E*-2-octenal, *Z*- and *E*-citral, 1-hydroxy-2-methoxy-benzene, benzeneethanol, β-pinene, *E*-2-decenal, *E*,*Z*-2,4-decadienal, *R*-limonene, geranyl acetone, and β-cyclocitrylideneacetone, were found, which have already been described in studies regarding the aroma compounds of tomato volatiles [30,31,33,34]. Additionally, some compounds were found that have not been linked to tomato volatiles to date. Our study adds some new compounds to the list of cherry tomato volatile compounds (such as 3-methyl-2-butenal, heptenal, *Z*-4-heptenal,*E*-2-heptenal, *E*-carveol, verbenol, limonene oxide, 2-decen-1-ol, *Z*-4-decen-1-al, caryophyllene oxide, and *E*,*E*-2,4-dodecadienal) that may better describe the cherry tomatoes’ volatile compounds.

A new approach was used to validate the identification result. Lower ionization energy allows us to find the molecular ion [M^+^] of the compounds on the mass spectrum, which can help to strengthen (validate) the NIST library match accuracy. Of the 59 identified compounds, 21 compounds could be confirmed by this method.

Agglomerative cluster analysis (CA) grouped the samples successfully, while linear discriminant analysis with principal component analysis (PCA-LDA) was used as a supervised method. Agglomerative cluster analysis dendrogram showed that the majority of the samples had similarities at a low level; therefore, these results suggest that the classification of the samples is possible, and their volatile compositions have distinct patterns. On the other hand, PCA-LDA provided an acceptable model, and the samples were sorted into different groups; therefore, the separation was successful. As a result, evaluating only 16 compounds instead of 64 is enough for successful separation.

## 4. Materials and Methods

### 4.1. Samples and Sampling Method

Eleven types of cherry tomatoes available on the market in Hungary were analyzed (Tesco Extra, Orange, Zebra, Yellow, Round Netherland, Mini San Marzano, Spar truss, Tesco Sunstream, Paprikakertész, Mc Dreamy, and Tesco Eat Fresh). All cherry tomato samples were first-class quality and had a generally unique appearance. The most important descriptive data for the samples have been already published in a separate paper [37]. As the first step of sample preparation, the samples were mashed and homogenized with a food chopper (Russell Hobbs 24662-56, Schlosser Kft., Budapest, Hungary), then a 5 mL homogenized sample was pipetted into a 10 mL headspace (HS) vial. The vials were immediately closed with a PTFE/silicone septum in order to preserve tomato aromas, and the equilibrium between the headspace and tomato samples was set at room temperature.

Sampling was carried out with the solid-phase microextraction (SPME) sampling technique. The proper SPME fiber and sampling parameters were optimized. The proper sample extraction time was defined as 30 min, while the sampling temperature was 60 °C. A semi-automatic shrink water bath (Saumya Technocrates Private Limited, Ahmedabad, Gujarat, India) was used to adjust the sampling temperature. SPME desorption was 4 min at 290 °C in the GC injector in a splitless mode. All measurements were carried out in triplicates.

### 4.2. Analytical Measurements

An Agilent 6890 gas chromatography (Santa Clara, CA, USA) coupled with an Agilent 5975 MSD mass spectrometer (Santa Clara, CA, USA) was used to analyze the extracted volatiles. High-purity hydrogen (purity: 99.9999%) was used as a carrier gas with a flow of 1.2 mL/min. An Agilent HP-5MS (Santa Clara, CA, USA) ((5%-phenyl)-methylpolysiloxane) 30.0 m × 250 µm × 0.25 µm) chromatographic column was used to separate the volatiles. The linear oven temperature program was used, where the initial temperature was 40 °C, then the temperature was increased by 10 °C/min until it reached 300 °C. The temperature was held at 300 °C for 5 min. The temperature of the transferline (which connects the GC to the MS) was 300 °C, the apt final column temperature in order to prevent possible condensation.

The MS source temperature was set to 230 °C and the quadrupole temperature was held at 150 °C. Positive electron ionization (EI+) was used, with an electron energy level of 70 eV. The detector was used at a scan mode in the range of 33–500 *m*/*z*. The MS was tuned using perfluorotributylamine (PFTB) every day before performing the measurements. Agilent Enhanced MSD ChemStation B.03.02 SR2 software handled the GC and MS parameters.

Agilent MassHunter Workstation Qualitative Analysis B.08.00 software was used for the evaluation and comparison of the chromatograms. The Agilent NIST 2017 Mass Spectral Library was used for compound identification, and two other libraries were also used to verify the identification results (W9N08 and W10N11).

### 4.3. Data Analysis

Cluster analysis (CA) was used as an unsupervised technique to test if there was any pattern in the dataset that grouped the mashed tomato samples based on their volatile patterns. Cluster analysis used an input data matrix that had 33 measurements (11 samples with 3 parallels) in the rows and 64 volatiles in rows. The data matrix contained the areas under the curve for all the volatiles. CA was run using the Euclidean distance and Ward’s method after a careful evaluation of multiple distance measurements and agglomeration schedules as suggested by [35].

As a supervised method, a linear discriminant analysis was run on scores using principal component analysis (PCA-LDA) [36]. PCA-LDA enabled the classification of the mashed tomato samples based on their volatile compounds. PCA was run on the original input matrix and tested using Kaiser–Meyer–Olkin (KMO) and Bartlett’s tests. Data analysis was performed using XL-Stat ver. 2023.2.1414.

## Figures and Tables

**Table 1:** List of volatile compounds from all tomato samples. The compound name is written according to the NIST library. RI: retention index, MW: molecular weight, ID: identification match factor according to the NIST library (%), n.d.: no data. Odor characteristic is described according to [32], indicated by ″^1^″; some aroma compounds were found by [31] and are indicated by ″^2^″.

#	RI	Compound Name	Formula	CAS	MW	ID (%)	Odour Characteristic
1	692	3-methyl-2-butenal	C_5_H_8_O	107-86-8	84.1	85.2	^1^ sweet, fruity, “green” hazelnut fragrance with cherry
2	806	1-hexanal	C_6_H_12_O	66-25-1	100.1	98.9	^1^ freshly cut grass, wood, citrus (“green” aroma group)
3	814	2-hexenal	C_6_H_10_O	505-57-7	98.1	91.8	^1^ fruity, fresh, friss, green herb, yeasty
4	814	(*E*)-2-hexenal	C_6_H_10_O	6728-26-3	98.1	97.8	^2^ fresh green aroma, grape
5	861	(*E*)-3-hexen-1-ol	C_6_H_12_O	928-96-1	100.1	92.0	^1^ fresh, raw fruity
6	860	1-hexanol	C_6_H_14_O	111-27-3	102.1	94.4	^1^ fruity, apple peel and oil
7	913	(*Z*)-4-heptenal	C_7_H_12_O	6728-31-0	112.1	84.9	
8	905	heptanal	C_7_H_14_O	111-71-7	114.1	91.8	
9	822	(*E*,*E*)-2,4-hexadienal	C_6_H_8_O	142-83-6	96.1	91.6	^1^ sweetish, green, waxy, aldehydic
10	913	(*E*)-2-heptenal	C_7_H_12_O	18829-55-5	112.1	90.2	
11	938	6-methyl-5-hepten-2-one	C_8_H_14_O	110-93-0	126.1	96.2	^1^ musty, apple, banana, green bean
12	1120	(*E*,*E*)-2,4-nonadienal	C_9_H_14_O	5910-87-2	138.1	88.4	^1^ cucumber scent
13	868	3(*E*)-3-ethyl-2-methyl-hexa-1,3-diene	C_9_H_16_	61142-36-7	124.1	84.5	
14	1067	2-isobutylthiazole	C_7_H_11_NS	18640-74-9	141.1	95.3	^2^ spicy tomato characteristic
15	1081	phenylacetaldehyde	C_8_H_8_O	122-78-1	120.1	90.4	^1^ flower, honey, tobacco, tropical fruity
16	905	1-vinyl-2,3-4,5-diepoxycyclohexane	C_8_H_10_O_2_	53966-43-1	138.1	85.4	
17	1228	(2*E*)-2,7-dimethyl-2,6-octadien-1-ol	C_10_H_18_O	22410-74-8	154.1	85.1	
18	1013	(*E*)-2-octenal	C_8_H_14_O	2548-87-0	126.1	95.0	^2^ waxy, green, leafy and mouldy
19	n.d.	1-oxacyclopropyl-3,4-epoxycyclohexane	C_8_H_12_O_2_	2000054-27-0	140.1	87.4	
20	n.d.	(*E*)-carveol	C_10_H_16_O	99-48-9	152.1	85.4	
21	n.d.	4-methylbenzaldehyde	C_8_H_8_O	104-87-0	120.1	80.0	
22	1174	(*Z*)-citral	C_10_H_16_O	106-26-3	152.1	85.0	
23	1090	1-hydroxy-2-methoxy-benzene	C_7_H_8_O_2_	90-05-1	124.1	93.2	^1^ woody, smoky, phenolic, bacon
24	1228	2(*E*)-2,7-dimethyl-2,6-octadien-1-ol	C_10_H_18_O	22410-74-8	154.1	82.4	
25	869	7-methyl-3-octyne	C_9_H_16_	37050-06-9	124.1	84.6	
26	1136	benzeneethanol	C_8_H_10_O	60-12-8	122.1	93.7	^1^ mushroom and rose blossom, with a sweetish scent
27	1138	benzyl nitrile	C_8_H_7_N	140-29-4	117.1	85.1	
28	1128	limonene dioxide	C_10_H_16_O_2_	96-08-2	168.1	85.3	
29	1136	verbenol	C_10_H_16_O	473-67-6	152.1	80.4	
30	1212	(*Z*)-4-decen-1-al	C_10_H_18_O	21662-09-9	154.1	87.2	
31	1266	2-decen-1-ol	C_10_H_20_O	18409-18-2	156.2	88.8	
32	n.d.	beta-pinene	C_10_H_16_	127-91-3	136.1	82.4	^1^ fresh pine, resinous, slightly spicy, camphorous scent
33	964	3,10-dioxatricyclo[4.3.1.0(2,4)]dec-7-ene	C_8_H_10_O_2_	2000050-41-5	138.1	82.7	
34	1204	1,3,4-trimethyl-3-cyclohexen-1-carboxaldehyde	C_10_H_16_O	40702-26-9	152.1	91.6	
35	1215	3-methyl-3-(4-methyl-3-pentenyl)-2-oxiranecarbaldehyde	C_10_H_16_O_2_	16996-12-6	168.1	85.7	
36	1174	(2*Z*)-3,7-dimethyl-2,6-octadienal	C_10_H_16_O	106-26-3	152.1	91.2	
37	n.d.	(*E*)-2-decenal	C_10_H_18_O	3913-81-3	154.1	82.4	^1^ waxy, earthy, coriander and mushroom with a chicken and pork fat scent
38	1174	(*E*)-citral	C_10_H_16_O	141-27-5	152.1	90.3	
39	n.d.	(*E*,*Z*)-2,4-decadienal	C_10_H_16_O	25152-83-4	152.1	87.4	^1^ herbal leaves and vegetable character
40	n.d.	2-phenylnitroethane	C_8_H_9_NO_2_	2000074-00-7	151.1	85.5	
41	904	2H-1b,4-ethanopentaleno[1,2-b]oxirene,hexahydro-, (1aa,1bb,4b,4aa,5aa)- (9CI)	C_10_H_14_O	117221-80-4	150.1	92.8	
42	1418	(*E*,*E*)-2,4-dodecadienal	C_12_H_20_O	21662-16-8	180.2	86.2	^1^ green, plant, waxy and aldehydic scent
43	1331	isobutyl 3-hydroxy-2,2,4-trimethylpentanoate	C_12_H_24_O_3_	244074-78-0	216.2	81.5	
44	1331	2,2,4-trimethyl-3-hydroxypentyl isobutyrate	C_12_H_24_O_3_	74367-34-3	216.2	82.7	
45	1249	R-limonene	C_10_H_16_O_3_	2000154-07-9	184.1	80.9	
46	1696	6(*E*),11(*E*)-6,11-tridecadienyl acetate	C_15_H_26_O_2_	2000319-55-7	238.2	81.5	
47	1326	2-(1-formylvinyl)-5-methylcyclopentanecarbaldehyde	C_10_H_14_O_2_	5951-57-5	166.1	84.5	
48	1381	hexyl caproate	C_12_H_24_O_2_	6378-65-0	200.2	80.0	^1^ sweet, fruity, with tropical character
49	1309	7-oxooctanoic acid	C_8_H_14_O_3_	14112-98-2	158.1	83.2	
50	1792	1,2-15,16-diepoxyhexadecane	C_16_H_30_O_2_	2000371-25-6	254.1	80.7	
51	n.d.	2(*E*)-3,7-dimethyl-2,6-octadien-1-ol	C_10_H_18_O	624-15-7	154.1	83.8	
52	1507	caryophyllene oxide	C_15_H_24_O	1139-30-6	220.2	85.3	^2^ woody, forest-like scent
53	1420	geranyl acetone	C_13_H_22_O	3796-70-1	194.2	92.4	^1^ floral, fruity, apple, banana
54	1457	beta-cyclocitrylideneacetone	C_13_H_20_O	14901-07-6	192.2	93.3	^1^ woody, sweetish, fruity, berry-like
55	1555	2,4-di-tert-butylphenol	C_14_H_22_O	96-76-4	206.2	89.1	
56	1641	2-methyl-4-(2,6,6-trimethylcyclohex-1-enyl)but-2-en-1-ol	C_14_H_24_O	62924-17-8	208.2	82.9	
57	1428	(*E*,*Z*)-6,10-dimethyl-3,5,9-undecatrien-2-one	C_13_H_20_O	13927-47-4	192.2	86.0	
58	1733	3-hydroxydodecanoic acid	C_12_H_24_O_3_	1883-13-2	216.2	80.0	
59	1752	4(*E*)-1,5,9-trimethyl-1-vinyl-4,8-decadienyl formate	C_16_H_26_O_2_	2000358-23-1	250.2	81.7	
60	n.d.	unknown1	found in all tomato samples with different intensity values				
61	n.d.	unknown2					
62	n.d.	unknown3					
63	n.d.	unknown4					
64	n.d.	unknown5					

**Table 2:** Validated identification using the 10 eV electron energy level.

RT (Min)	Validated Compound Name	Formula	Molecular Weight	Molecular Ion 5 eV
3.8	hexanal	C_6_H_12_O	100.1	100.1
5.3	2-hexenal	C_6_H_10_O	98.1	98.1
7.4	(*E*,*E*)-2,4-hexadienal	C_6_H_8_O	96.1	96.1
11.2	6-methyl-5-heptene-2-one	C_8_H_14_O	126.1	126.1
13.1	(3*E*)-3-ethyl-2-methyl-hexa-1,3-diene	C_9_H_16_	124.1	124.1
13.2	2-isobutylthiazole	C_7_H_11_NS	141.1	141.2
13.7	phenylacetaldehyde	C_8_H_8_O	120.1	120.9
15.5	4-methylbenzaldehyde	C_8_H_8_O	120.1	119.9
16.0	guaiacol	C_7_H_8_O_2_	124.1	124.0
16.9	7-methyl-3-octyne	C_9_H_16_	124.1	124.0
17.2	1-phenyl-2ethanol	C_8_H_10_O	122.1	122.0
18.4	phenylacetonitrile	C_8_H_7_N	117.1	117.1
21.8	beta-pinene	C_10_H_16_	136.1	135.9
22.2	1,3,4-trimethyl-3-cyclohexenyl-1-carboxaldehyde	C_10_H_16_O	152.1	152.1
24.4	*E*-citral	C_10_H_16_O	152.1	151.9
25.8	2*H*-1b,4-ethanopentaleno[1,2-b]oxirene, hexahydro-, (1a.alpha.,1b.beta.,4.beta.,4a.alpha.,5a.alpha.)	C_10_H_14_O	150.1	151.1
29.7	geranyl acetone	C_13_H_22_O	194.2	194.1
30.0	deisopropylatrazine	C_5_H_8_ClN	173.0	173.0
30.3	beta-ionone	C_13_H_20_O	192.2	192.0
30.9	2,4-di-tert-butylphenol	C_14_H_22_O	206.2	206.0
32.0	(3*E*,5*Z*)-6,10-dimethylundeca-3,5,9-trien-2-one	C_13_H_20_O	192.2	192.0

## Data Availability

The raw data supporting the conclusions of this article will be made available by the authors upon request.
